# Enhanced Broadband Electromagnetic Absorption in Silicon Film with Photonic Crystal Surface and Random Gold Grooves Reflector

**DOI:** 10.1038/srep12794

**Published:** 2015-08-04

**Authors:** Zhi-Hui Chen, Na Qiao, Yibiao Yang, Han Ye, Shaoding Liu, Wenjie Wang, Yuncai Wang

**Affiliations:** 1 Key Lab of Advanced Transducers and Intelligent Control System, Ministry of Education and Shanxi Province, College of Physics and Optoelectronics, Taiyuan University of Technology, Taiyuan 030024, China; 2 State Key Laboratory of Information Photonics and Optical Communications, Beijing University of Posts and Telecommunications, Beijing 100876, China

## Abstract

We show a hybrid structure consisting of Si film with photonic crystal surface and random triangular gold grooves reflector at the bottom, which is capable of realizing efficient, broad-band, wide-angle optical absorption. It is numerically demonstrated that the enhanced absorption in a broad wavelength range (0.3–9.9 μm) due to the scattering effect of both sides of the structure and the created resonance modes. Larger thickness and period are favored to enhance the absorption in broader wavelength range. Substantial electric field concentrates in the grooves of surface photonic crystal and in the Si film. Our structure is versatile for solar cells, broadband photodetection and stealth coating.

Efficient broadband electromagnetic absorption is significant in solar energy harvesting^1^,^2^, infrared photodetection^3^, and stealth coating^4^.

In 1982, Yablonovitch limit (Y-limit) was proposed to show a light trapping limit in homogeneous semiconductor films^5^. However, it is only applicable to geometrical optics and could be surpassed by the light trapping in sub-wavelength nanostructures by combining the reflection, diffraction, and refraction effects. A lot of optical coupling structures have been designed to enhance the electromagnetic absorption^2^,^3^,^6^,^7^,^8^,^9^,^10^,^11^,^12^,^13^,^14^,^15^,^16^,^17^,^18^,^19^,^20^,^21^,^22^, and various optimized light trapping structures are explored by optimization algorithms^23^,^24^. Most of these structures acted on either visible wavelength range or infrared wavelength range. However, simultaneously large absorption in both visible and infrared wavelength ranges is also essential for solar cells, broadband photodetectors and stealth applications.

In this work, we propose a Si film with one dimensional (1D) photonic crystal (PC) on the surface and 1D random triangular gold grooves reflector (RTGGR) at the bottom to improve the electromagnetic absorption efficiency within the wavelength range from 0.3 μm to 9.9 μm. The surface PC is capable of reducing the reflection from the interface and increasing the optical path lengths in the Si film. Meanwhile, enhanced scattering and absorption of light is obtained by introducing RTGGR at the bottom. The absorption is close to the Y-limit at visible wavelengths and is also large in infrared wavelength range, especially for the transverse magnetic (TM) polarized light. Furthermore, the enhanced absorption efficiency is insensitive to the incidence angle.

In the following, we take ultrathin silicon solar cell as an example, and the wavelength range of 0.3–2.0 μm is studied firstly. The cross-sectional view of the proposed structure with denoted geometrical parameters is shown in Fig. 1(a). The calculations are conducted within one period by using rigorous coupled wave analysis (RCWA)^25^,^26^ and finite difference time domain (FDTD) method^3^,^27^,^28^. The model parameters and calculation details can be found in Method section.

## Results and Discussion

### Effects of period and angle on the absorption spectr**a**

**Figure 1:**
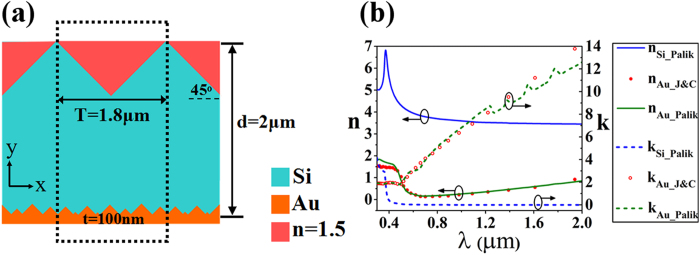
(**a**) The cross-sectional views of the proposed structure. One period is shown in dotted region. Polymer can be filled in the surface PC (n = 1.5). (**b**) The refractive indices of Si and gold in the wavelength range from 0.3 μm to 2 μm.

**Figure 2:**
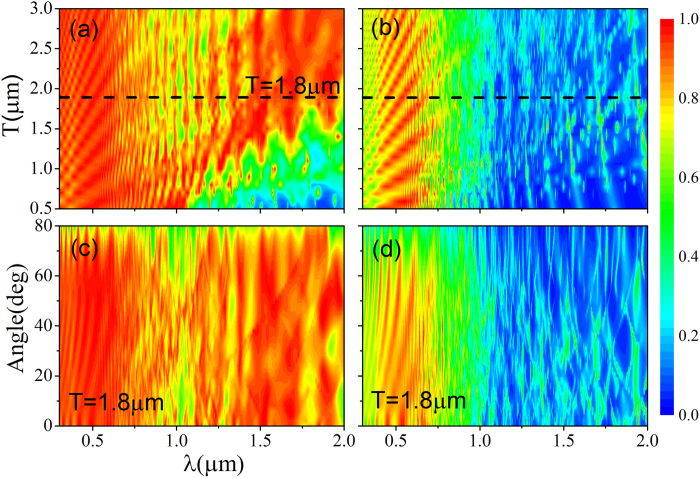
(**a,b**) are the absorption efficiencies of TM and TE polarization at normal incidence respectively when T changes from 0.5 μm to 3.0 μm. Figure 2 (**c,d**) are the absorption efficiencies of TM and TE polarization respectively at T = 1.8 μm when the incidence angle changes from 0° to 80°.

Figure 2 (a,b) displays the effect of period (T) of our structure (Fig. 1(a)) on the absorption efficiency at TM and TE polarization, respectively. It is observed that for TM polarization, the mean absorption efficiency of the structure exceeds 70% within the whole wavelength range from 0.3 μm to 2 μm when T is larger than 1.7 μm. The high absorption efficiencies of TE polarization are within the wavelength range from 300 nm to 700 nm. According to Fig. 2(a), the optimal periodicity should be close to the near-infrared wavelength for enhancing the electromagnetic absorption of TM polarization in both visible and near-infrared wavelength range. This is because for longer wavelength, a larger period is preferred to create more guided resonances which lead to higher absorption. In the following study, we choose T = 1.8 μm.

Figure 2(c,d) shows the angular absorption spectra of TM and TE polarization in our proposed structure. It is confirmed that the absorption of structure is insensitive to the angular changes when the incidence angle changes from 0° to 80°.

### Effects of coupling structures on the absorption spectra

**Figure 3:**
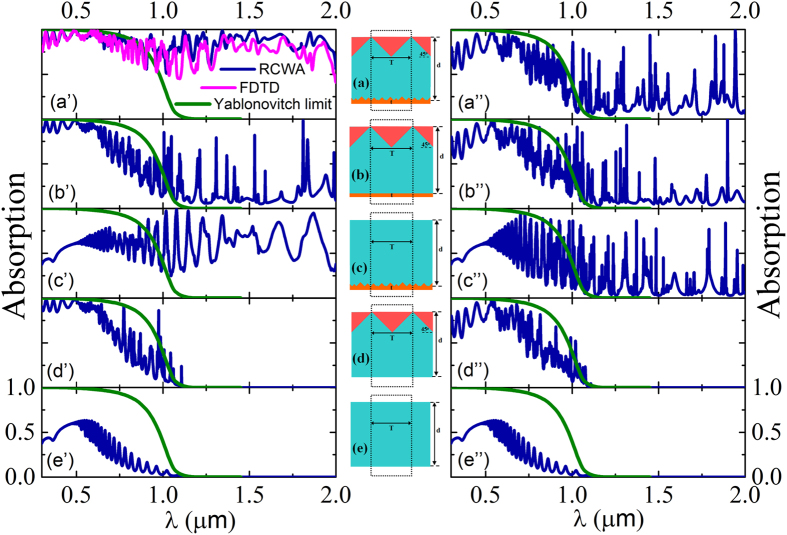
Absorption spectra of (a’–e’) TM and (a”–e”) TE polarizations at normal incidence in five structures (a–e) when T = 1.8 μm. In all subfigures, blue curves are obtained by RCWA method, green curves are Y-limit, the pink curve in (**a’**) is obtained by FDTD method.

In order to investigate the physical mechanism of light absorption enhancement, the absorption spectra of five structures compared with the Y-limit are shown in Fig. 3.

From Fig. 3(a’), we can see that the result from RCWA method is in accordance with that from FDTD method. The Y-limit with perfect antireflection and perfect light trapping is calculated by:


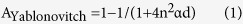


which is shown in green curves^2^. (“n” is the real part of the refractive index of Si, “α” is the absorption coefficient, “d” is the thickness of Si film.)

When there is a bare Si film (structure “e”), the absorption is much smaller than the Y-limit. When only the surface PC is introduced (structure “d”), the absorption is enhanced due to the scattering and diffraction, but it is still below the Y-limit. When only the RTGGR is added (structure “c”), the absorption at infrared wavelengths is enhanced a lot, but the absorption at the visible wavelengths is not large. When both the surface PC and flat gold reflector are added (structure “b”), the absorption at infrared wavelengths is large at individual wavelengths due to some high Q-factor resonance modes. When both surface PC and RTGGR are introduced (structure “a”), obvious optical absorption enhancement is observed at both visible and infrared bands, and the absorption spectrum of our structure is very close to the Y-limit at visible wavelength and the absorption can even maintain large within infrared wavelengths range. It is noted that the absorption efficiencies of TM polarization in our proposed structure (structure “a”) are higher than other four structures, especially in infrared wavelength range, the high absorption bands cover both visible and infrared bands. Similarly, gold reflector can also enhance the absorption of TE polarization at infrared wavelengths, while the enhancement of TE polarization in our structure is not as good as TM polarization, which is due to the 1D PC surface and 1D gold grooves. The surface PC and RTGGR work together on visible and infrared bands. Whether the top surface of Si is flat or structured, there will be resonance modes between the top surface and gold reflector. Stronger resonance modes can lead to more absorption by the Si and gold material. We show the electric field distributions corresponding to individual absorption peaks (TE polarization) of the structures (a–c) in Fig. S1 of the Supplementary information file. There are no individual peaks in absorption spectra in the infrared wavelength range for structure (d) and (e) because they do not contain gold film and the Si film has little absorption in the infrared wavelength range.

### Spatial distributions of the electric field

**Figure 4:**
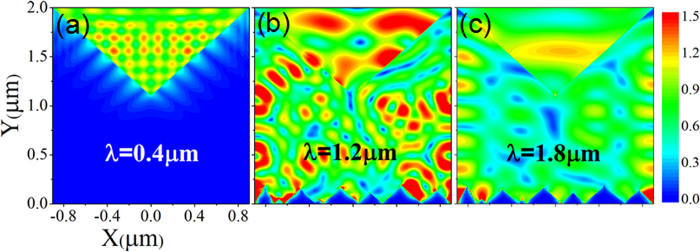
Electric field distribution (E^2^) of TM polarization at (**a**) λ_1_ = 0.4 μm, (**b**) λ_2_ = 1.2 μm, and (**c**) λ_3_ = 1.8 μm, respectively, in the structure of Fig. 1(a).

In order to study the spatial distributions of electric field in the proposed structure (Fig. 1(a)), we focused on TM polarization. Figure 4 displays distribution of the electric field (**E**^2^ = E_x_^2^ + E_y_^2^ + E_z_^2^) calculated by RCWA at wavelengths of λ_1_ = 0.4 μm, λ_2_ = 1.2 μm, and λ_3_ = 1.8 μm, respectively. It can be seen from Fig. 4 that the electric field enhancement mainly concentrates in triangular grooves of surface PC at short wavelength. And at longer wavelength, the electric field distributes in both triangular grooves of surface PC and the Si film between PC and gold reflector. The distribution of the electric field has been confirmed by FDTD method. In solar cells or other photovoltaic applications, different type or different size of colloidal quantum dots (QDs) can be embedded in the PC’s grooves to further enhance the broadband absorption^29^, because QDs have broadband absorption from the UV to the visible and even into the infrared wavelength regions mainly depending on the type and size of QDs^30^,^31^,^32^. Additionally, the optical resonance modes in the PC’s grooves will enhance the interaction between light and QDs. When QDs are introduced into the surface PC’s grooves, the increase of absorption and the wavelengths for the absorption enhancement towards the whole structure are related to the refractive index (n) and extinction coefficient (k) of the surface PC’s grooves region, which are depended on the type, size and concentration (volume fraction ratio) of the QDs, and the QDs are usually in the status of film in a device^33^. In order to estimate the increase of absorption after embedding QDs in the grooves, we take (PbSe)_85_Cd_15_ QD as an example, we calculated the absorption efficiency (at TE polarization) of the structure with (PbSe)_85_Cd_15_ QDs film embedded in the surface PC’s grooves (please see the model in Fig. S2 of the Supplementary information file). The refractive index (n = 1.7) and extinction coefficient (k = 0.42) of (PbSe)_85_Cd_15_ QDs thin film are obtained at the wavelength of λ = 885 nm (*hν* = 1.4 eV)^34^. According to the RCWA calculation results, the absorption efficiency of our proposed structure with polymer film (n = 1.5, k = 0) in the surface PC’s grooves is about 44.3%, when (PbSe)_85_Cd_15_ QDs thin film (n = 1.7, k = 0.42) is introduced into the surface PC’s grooves, the absorption efficiency of the structure can be enhanced to 92.6%.

### Time resolved electric field

We set three point monitors which are positioned in the surface PC’s groove, Si film far from gold, Si film near the gold, respectively, in structure “a” and structure “e”. The fold of time integral **E**^2^ between structure “a” and structure “e” is defined as:


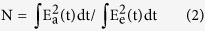


**Figure 5:**
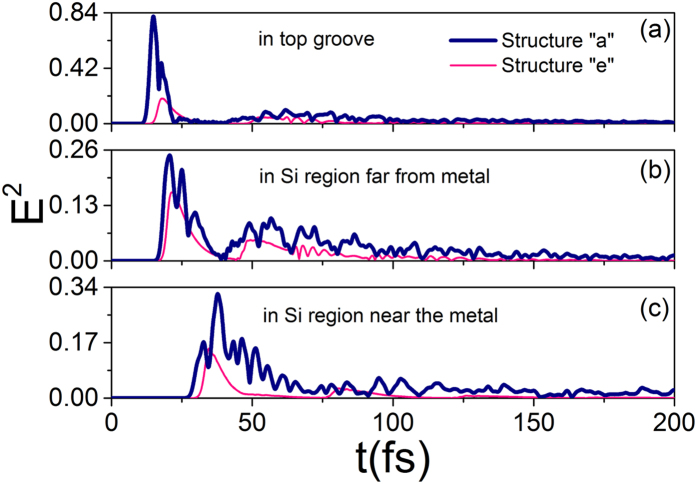
E^2^(t) in the surface PC’s groove, Si film far from metal, Si film near the metal in structure “**a**” **a**nd structure “**e**”. The source is a broadband pulse covering 0.3–2.0 μm.

The N is “3.6”, “2.6” and “4.7” in Fig. 5 (a–c), respectively. Time integral **E**^2^ in structure “a” is larger than that of structure “e”, which could confirm the results obtained in the frequency-domain (Fig. 3). The enhancement of electric field is because of light scattering and resonance modes in structure “a”.

### Enhanced broadband (0.3–9.9 μm) absorption

**Figure 6:**
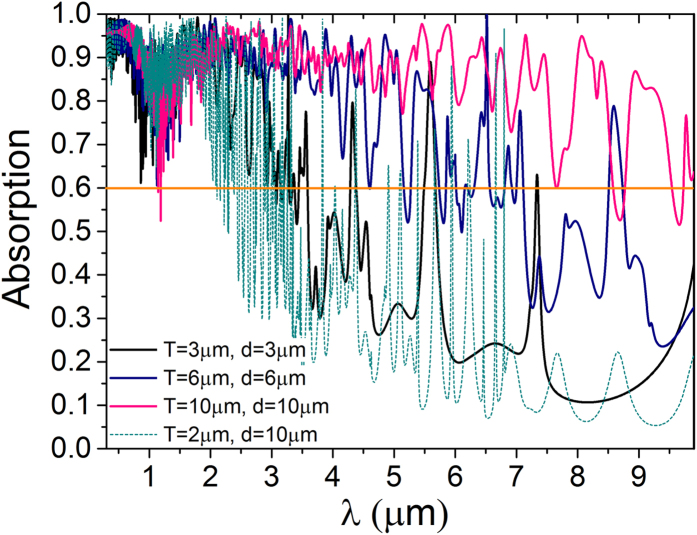
Absorption spectra of TM polarization at normal incidence in our structure with different periods (T) and thicknesses (d) of Si. The dark yellow line at 60% absorption is just for eye guide.

As the optimal periodicity for light trapping should be close to the target wavelength range, absorption enhancement in broader wavelength range can be realized by using larger thickness and period. From Fig. 6, we can see that the large absorption (above 60%) wavelength range is more related to the T than the d. For different T, the absorption is similar at shorter wavelength, while is different at longer wavelength, and the drop point of absorption is at around the wavelength near the value of the T. Large d can only result in more resonance modes than the small d. When T = 10 μm and d = 10 μm, the absorption is larger at the wavelength from 0.3 μm to 9.9 μm which covers both the visible wavelength and infrared wavelength compared to the other smaller T and d. In our structure, T can be even larger and hold more guided resonances without obvious optical coupling and leaking to external channels.

## Conclusions

In summary, we have numerically demonstrated a hybrid structure consisting of Si film with a photonic crystal surface and a random triangular gold groove reflector at the bottom, which is capable of realizing efficient (>60%), broad-band (0.3 μm–9.9 μm), wide-angle (0°–80°) electromagnetic absorption. It is observed that the absorption efficiencies of TM polarization in our proposed structure is close to Y-limit within the visible wavelength range and maintain large within the infrared wavelength range, such large absorption is insensitive to the incidence angle. Thus is mainly attributed to the enhancement of light scattering and light trapping in our structure. The surface PC and RTGGR work together on visible and infrared bands. The results are confirmed by the FDTD method and RCWA method. Colloidal quantum dots can be embedded in the surface PC’s grooves to further enhance the absorption. The multifunctional structure provides a great potential for the application in solar cells, photodetectors and stealth coating.

## Methods

### Proposed structure

In the whole study, the calculations are conducted within one period (the dotted region) as shown in Fig. 1(a). The period (T) of the surface PC is 1.8 μm, and the cross-sectional views of one period of the PC is isosceles right triangle. We use eight isosceles right triangles with random sizes in sub-wavelength scale in one period to represent the quasi-random distribution of gold grooves. The thickness of Si (d) is 2 μm, and the thickness of gold substrate (t) is 100 nm which is thick enough to reflect the light in the wavelength range from 0.3 μm to 9.9 μm. The absorption efficiencies of TM and TE polarization are studied when T and incidence angle change. Here, the size of every gold triangle is in a certain proportion to T. The results are confirmed by the RCWA method and FDTD method.

### RCWA and FDTD simulations

The Rigorous Coupled Wave Analysis (RCWA) method, which is an efficient method to calculate electromagnetic wave propagation in periodic structures^26^, is adopted in our work. In this method, Maxwell’s equations are solved in the Fourier domain. The absorption can be calculated by the reflected, transmitted and diffracted power. In this work, the absorption efficiency has been computed in one unit of the periodic structure within the space domain (x, y) = (−0.9:0.9, −1.0:4.5) μm, the wavelength, incidence angle and period are scanned. The near-field electric field distributions are calculated by using a normal-direction incident plane wave source at specific wavelengths.

The finite-difference time-domain (FDTD) method can numerically solve two or three dimensional time-dependent Maxwell’s equations in time domain without any approximations or theoretical restrictions^35^. In addition, FDTD can also obtain frequency-dependent solutions by using fast Fourier transform (FFT) or discrete Fourier transform (DFT). Thus the transmission and reflection of light can be calculated, the absorption can be obtained according to the law of energy conservation. In this work, we perform a two dimensional FDTD simulation in the region (x, y) = (−0.9:0.9, −1.0:4.5) μm with Bloch boundaries in the x direction and perfectly matched layers (PMLs) absorbing boundaries in the y direction. The simulation time is 20000 fs, and the spatial grid size is 4nm. The incident plane wave is simulated by a broadband pulse.

In both simulation methods, we take intrinsic losses of Si and gold into consideration. In the RCWA method and FDTD method, the wavelength dependent refractive indices of Si are obtained from Palik’s book^36^, while the refractive indices of gold are from Palik’s book^36^ and Johnson and Christy’s data^37^ respectively. The complex refractive index of Si and gold are shown in Fig. 1(b). Plane wave is injected from the top of the structure, and the wavelength range we studied firstly in this work is from 0.3 μm to 2.0 μm. Then a broader band (0.3 μm–9.9 μm) is studied.

### Feasibility of experimental realization

Our structure is experimentally feasible. The grooves in the upper and bottom surface of Si film can be fabricated by various etching techniques^38^,^39^. Then the grooves at two sides of Si can be filled by deposited polymer and gold film, respectively. The structure is expected to enhance both TM and TE polarization obviously if it includes 2D patterned grooves in the upper and bottom surface of the Si film.

## Additional Information

**How to cite this article**: Chen, Z.-H. *et al.* Enhanced Broadband Electromagnetic Absorption in Silicon Film with Photonic Crystal Surface and Random Gold Grooves Reflector. *Sci. Rep.* **5**, 12794; doi: 10.1038/srep12794 (2015).

## Supplementary Material

Supplementary Information
