# Supplementary material for: Enhanced Broadband Electromagnetic Absorption in Silicon Film with Photonic Crystal Surface and Random Gold Grooves Reflector
Source: Sci Rep. 2015 Aug 4;5:12794. doi: 10.1038/srep12794 (PMC4536523; doi:10.1038/srep12794)
Supplement: Supplementary Information [file srep12794-s1.pdf]

Enhanced Broadband Electromagnetic Absorption in Silicon Film with Photonic Crystal Surface and Random Gold Grooves Reflector

Zhi-Hui Chen, [1,2,*,‡] Na Qiao, [1,‡] Yibiao Yang, [1] Han Ye, [2] Shaoding Liu, [1] Wenjie Wang, [1] and Yuncai Wang [1]

[1] *Key Lab of Advanced Transducers and Intelligent Control System, Ministry of Education and Shanxi Province, College of Physics and Optoelectronics, Taiyuan University of Technology, Taiyuan 030024, China.*

[2] *State Key Laboratory of Information Photonics and Optical Communications, Beijing University of Posts and Telecommunications, Beijing 100876, China*

*Correspondence and requests for materials should be addressed to: Z.H.C. (huixu.chen@gmail.com)

‡Zhi-Hui Chen and Na Qiao contributed equally to this work.

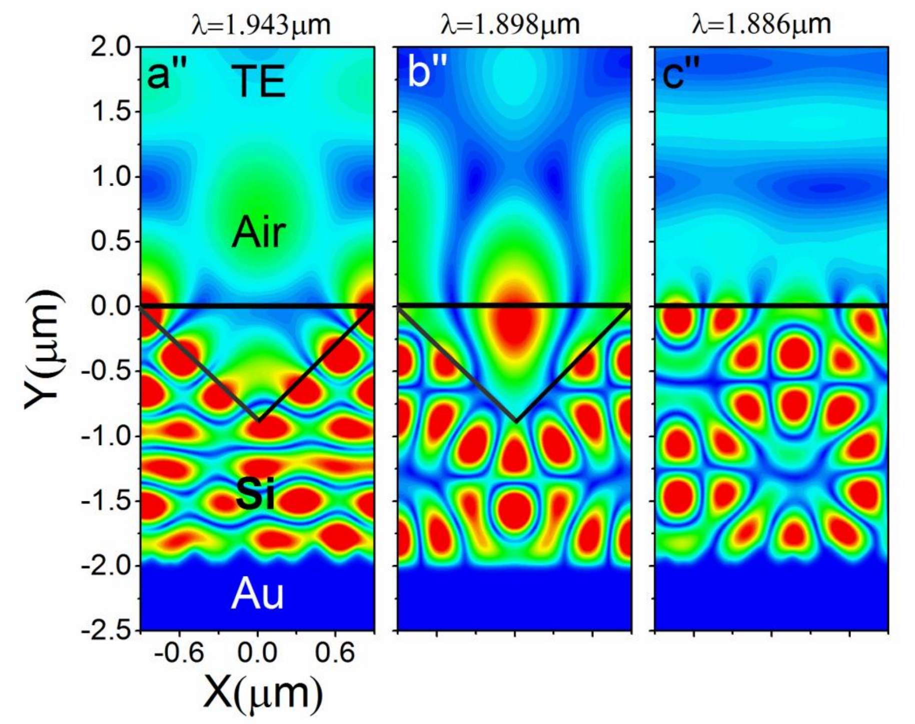

Figure S1 | (a''-c'') Electric field distributions at TE polarization corresponding to the absorption peaks of the structure (a-c).

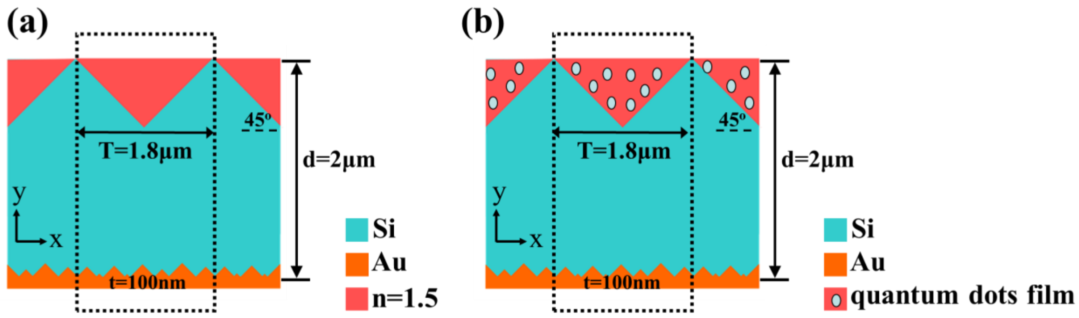

Figure S2 | (a) Proposed structure in the manuscript, (b) Quantum dots embedded in the surface PC's grooves
